# Higher intake of whole grains and dietary fiber are associated with lower risk of liver cancer and chronic liver disease mortality

**DOI:** 10.1038/s41467-021-26448-9

**Published:** 2021-11-04

**Authors:** Xing Liu, Wanshui Yang, Jessica L. Petrick, Linda M. Liao, Weibing Wang, Na He, Peter T. Campbell, Zuo-Feng Zhang, Edward Giovannucci, Katherine A. McGlynn, Xuehong Zhang

**Affiliations:** 1 Department of Nutrition, T.H. Chan School of Public Health, Harvard University, Boston, MA USA; 2 Department of Epidemiology, School of Public Health, Fudan University, Shanghai, P.R. China; 3 Channing Division of Network Medicine, Department of Medicine, Brigham and Women’s Hospital and Harvard Medical School, Boston, MA USA; 4 Department of Nutrition, School of Public Health, Anhui Medical University, Hefei, Anhui P.R. China; 5 Slone Epidemiology Center, Boston University, Boston, MA USA; 6 Division of Cancer Epidemiology and Genetics, National Cancer Institute, Bethesda, MD USA; 7 Behavioral and Epidemiology Research Group, American Cancer Society, Atlanta, GA USA; 8 Department of Epidemiology, Fielding School of Public Health, University of California, Los Angeles, CA USA; 9 Jonsson Comprehensive Cancer Center, UCLA, Los Angeles, CA USA; 10 Department of Epidemiology, T.H. Chan School of Public Health, Harvard University, Boston, MA USA

**Keywords:** Nutrition disorders, Hepatocellular carcinoma, Lifestyle modification, Epidemiology

## Abstract

The relationship between dietary factors and liver disease remains poorly understood. This study evaluated the associations of whole grain and dietary fiber intake with liver cancer risk and chronic liver disease mortality. The National Institutes of Health–American Association of Retired Persons Diet and Health Study cohort recruited 485, 717 retired U.S. participants in 1995–1996. Follow-up through 2011 identified 940 incident liver cancer cases and 993 deaths from chronic liver disease. Compared with the lowest, the highest quintile of whole grain intake was associated with lower liver cancer risk (Hazard ratio [HR]_Q5 vs. Q1_ = 0.78, 95% confidence interval [CI]: 0.63–0.96) and chronic liver disease mortality (HR_Q5 vs. Q1_ = 0.44, 95% CI: 0.35–0.55) in multivariable Cox models. Dietary fiber was also associated with lower liver cancer risk (HR_Q5 vs. Q1_ = 0.69, 95% CI: 0.53–0.90) and chronic liver disease mortality (HR_Q5 vs. Q1_ = 0.37, 95% CI: 0.29–0.48). Fiber from vegetables, beans and grains showed potential protective effect. Here, we show that higher intake of whole grain and dietary fiber are associated with lower risk of liver cancer and liver disease mortality.

## Abstract (web summary)

Higher intake of dietary fiber and whole grains are associated with reduced risk of various diseases including some cancers. Here, the authors estimate reductions in liver cancer of 22% and 31% and chronic liver disease mortality of 56% and 63% associated with increased whole grain and dietary fiber intake, respectively.

## Introduction

Primary liver cancer is the sixth most common cancer and the third most common cause of cancer-related mortality worldwide^1^, and its incidence and mortality have been increasing in the United States^2^. The five-year survival rate of liver cancer has only improved from 11.7% to 21.3% during 2000–2011^3^, suggesting the importance of primary prevention for this fatal disease. Hepatocellular carcinoma (HCC) and intrahepatic cholangiocarcinoma (ICC) are the two most common histologic types of primary liver cancer, accounting for 75–80% and 10–15% of cases, respectively^4,5^. Major known risk factors for HCC include chronic hepatitis B virus (HBV) infection, chronic hepatitis C virus (HCV) infection, aflatoxin, heavy alcohol consumption, tobacco smoking, obesity, nonalcoholic fatty liver disease (NAFLD), and type 2 diabetes mellitus (T2DM)^6,7^. These factors are also associated, although not as strongly, with ICC. Certain biliary tract conditions, including choledochal cyst, choledocholithiasis, and cholelithiasis, are strongly associated with ICC^8^. Chronic liver diseases (CLD), including cirrhosis, fibrosis, alcoholic liver disease, and chronic hepatitis, are important precursors of HCC. Additionally, CLD may lead to death itself and is also a significant public health concern^9^. Non-HBV/non-HCV-related HCC is common in the U.S., with the etiology poorly understood^6^. More HCC and CLD are probably attributable to obesity, T2DM and NAFLD in this population^6^, which are closely associated with behaviors including dietary patterns and composites. Thus, identifying dietary factors that could reduce the risk of liver cancer and CLD mortality is of interest. However, to date, such research is limited^10,11^.

Whole grains contain the endosperm, as do refined grains, but also bran and germ. Thus, whole grains serve as valuable food sources of dietary fiber, vitamin B, E, selenium, zinc, copper, magnesium, and phytochemicals^12^. Whole grains and dietary fiber have long been considered beneficial in lowering the risks of T2DM^13,14^, cardiovascular diseases^15,16^, and some types of human cancers^17–20^. Intake of whole grains has also shown a potential beneficial impact on the risk or progression of NAFLD^21,22^. A recent systematic review suggested a protective role for whole grain intake in HCC risk^23^. So far, two prospective cohort studies have reported inverse associations between dietary fiber intake and liver cancer risk^24,25^, and one reported an inverse association for whole grain intake^25^. However, these two studies included a limited number of cases. Specifically, the European Prospective Investigation into Cancer and Nutrition (EPIC) included 191 HCC and 66 ICC, while the Nurses’ Health Study (NHS) and the Health Professionals Follow-up Study (HPFS) included 141 HCC cases. No study has yet examined the association between whole grain and dietary fiber intake and risk of CLD death.

In this work, we report inverse associations of whole grain and dietary fiber intake with both incident liver cancer and CLD deaths by utilizing the National Institutes of Health (NIH)–American Association of Retired Persons (AARP) Diet and Health Study with over 900 cases each.

## Results

### Baseline characteristics of the cohort participants

A total of 290,484 men (59.8%) and 195,233 (40.2%) women were included in this study. The mean age at study entry was 61.5 years old (SD = 5.4 y). The majority of the participants were white (92.8%), and 39.8% had, at least, a college degree or above. Participants in the highest quintile of whole grain intake or total dietary fiber intake compared with those in the lowest quintile were more physically active, had lower alcohol and tobacco consumption, and were more likely to have a self-reported history of diabetes (Table 1). Similar patterns were also observed for men (Supplementary Table 1) and for women (Supplementary Table 2).

**Table 1:** Baseline characteristics according to whole grain and total fiber intake among participants of National Institute of Health, American Association of Retired Persons (NIH–AARP) Diet and Health Study.

	Quintile categories for whole grain intake					Quintile categories for total dietary fiber intake				
	Quintile 1 (*n* = 96,681)	Quintile 2 (*n* = 96,245)	Quintile 3 (*n* = 99,242)	Quintile 4 (*n* = 96,150)	Quintile 5 (*n* = 97,399)	Quintile 1 (*n* = 97,179)	Quintile 2 (*n* = 97,139)	Quintile 3 (*n* = 97,095)	Quintile 4 (*n* = 97,189)	Quintile 5 (*n* = 97,115)
Age, yr	61.5 (5.4)	61.5 (5.4)	61.5 (5.4)	61.5 (5.4)	61.5 (5.4)	61.5 (5.4)	61.5 (5.4)	61.5 (5.4)	61.5 (5.4)	61.5 (5.4)
Female, %	40.2	40.0	40.0	40.5	40.3	40.2	40.2	40.2	40.2	40.2
White, %	91.9	92.6	93.4	93.6	92.2	92.1	93.7	93.8	93.4	90.8
College and above, %	33.7	39.1	41.4	43.0	41.6	34.9	38.7	40.8	41.9	42.6
BMI, kg/m^2^	27.1 (5.1)	27.2 (5.0)	27.1 (4.9)	27.0 (4.9)	26.9 (5.2)	27.1 (5.0)	27.1 (5.0)	27.1 (4.9)	27.1 (5.0)	27.0 (5.2)
Physical activity 5+ times/week, %	15.5	17.4	19.0	20.9	24.2	12.5	16.0	18.7	21.6	28.1
Alcohol, gram/day	14.7 (31.8)	11.8 (25.5)	10.7 (22.6)	9.9 (21.1)	8.1 (17.9)	12.7 (28.2)	11.7 (25.7)	11.4 (24.7)	10.6 (22.8)	8.9 (19.3)
Current smoking, %	19.2	12.9	10.9	9.4	8.7	19.2	13.6	11.1	9.6	7.5
Self-reported diabetes, %	7.4	8.4	8.8	9.2	11.1	7.8	8.5	9.0	9.4	10.3
Whole grains, oz/d	0.2 (0.1)	0.5 (0.1)	0.8 (0.1)	1.2 (0.2)	2.2 (0.9)	0.5 (0.3)	0.7 (0.5)	1.0 (0.6)	1.2 (0.7)	1.7 (1.1)
*Dietary fiber intake, types, and food sources, g/d*										
Total	13.2 (6.8)	16.0 (6.9)	18.2 (7.1)	20.9 (7.7)	26.4 (9.6)	9.1 (2.2)	13.7 (1.5)	17.5 (1.6)	22.0 (2.0)	32.5 (7.8)
Fruits	3.0 (3.0)	3.7 (3.0)	4.1 (3.1)	4.5 (3.2)	5.3 (3.8)	1.7 (1.2)	2.8 (1.7)	3.7 (2.1)	4.9 (2.6)	7.5 (4.5)
Vegetables	5.1 (3.5)	5.8 (3.5)	6.2 (3.6)	6.8 (3.9)	7.9 (4.6)	3.1 (1.3)	4.6 (1.7)	5.8 (2.1)	7.3 (2.6)	11.0 (5.1)
Beans	1.6 (2.0)	1.9 (2.1)	2.0 (2.2)	2.3 (2.4)	2.7 (3.0)	0.9 (0.8)	1.4 (1.1)	1.8 (1.4)	2.3 (1.9)	4.1 (4.0)
Grains	3.3 (2.2)	4.5 (2.3)	5.7 (2.3)	7.1 (2.5)	10.3 (3.7)	3.2 (1.5)	4.7 (1.9)	5.9 (2.3)	7.3 (2.9)	9.7 (4.5)

Values are means (SD) for continuous variables, percentages for categorical variables, and are standardized to the age distribution of the study population.

^*^Value is not age-adjusted.

### Whole grain, dietary fiber intake, and risk of liver cancer

After a median follow-up time of 15.5 years, a total of 940 participants developed primary liver cancer, including 635 HCC and 164 ICC. Whole grain intake was associated with a 22% risk reduction of liver cancer (HR_Q5 vs. Q1_ = 0.78, 95% CI: 0.63–0.96, *P*_*trend*_ = 0.01) after multivariable adjustment. Total dietary fiber intake was associated with 31% risk reduction (HR_Q5 vs. Q1_ = 0.69, 0.53–0.90, *P*_*trend*_ < 0.001) (Table 2). For different sources of fiber, significantly inverse associations were observed for vegetables (HR_Q5 vs. Q1_ = 0.65, 95% CI: 0.52–0.83, *P*_*trend*_ < 0.001) and grains (HR_Q5 vs. Q1_ = 0.78, 95% CI: 0.62–0.99, *P*_*trend*_ = 0.04), but not for fruits (HR_Q5 vs. Q1_ = 0.94, 95% CI: 0.76–1.17, *P*_*trend*_ = 0.82) or beans (HR_Q5 vs. Q1_ = 0.89, 95% CI: 0.71–1.10, *P*_*trend*_ = 0.02). The inverse associations for total dietary fiber and fiber from vegetables were observed for men (Supplementary Table 3), and to a less extent for women (Supplementary Table 4). Significant inverse associations were found between total dietary fiber and fiber from vegetables and risk of HCC (Supplementary Table 5), but not risk of ICC (Supplementary Table 6).

**Table 2:** The associations between whole grains and dietary fiber with risk of liver cancer from the NIH–AARP Diet and Health Study.

	HR (95% CI)						*P trend*
	Quintile 1	Quintile 2	Quintile 3	Quintile 4	Quintile 5	Per SD increase	
*Whole grains*							
Case number	214	194	198	155	179		
Model 1	1 (ref)	0.88 (0.72, 1.07)	0.86 (0.71, 1.04)	0.68 (0.56, 0.84)	0.77 (0.64, 0.95)	0.91 (0.84, 0.97)	0.007
Model 2	1 (ref)	0.91 (0.74, 1.10)	0.91 (0.75, 1.11)	0.73 (0.59, 0.90)	0.78 (0.63, 0.96)	0.90 (0.83, 0.97)	0.005
*Total dietary fiber*							
Case number	193	210	177	187	173		
Model 1	1 (ref)	1.06 (0.87,1.29)	0.88 (0.72,1.08)	0.92 (0.75,1.12)	0.84 (0.69,1.04)	0.90 (0.83,0.98)	0.016
Model 2	1 (ref)	1.04 (0.85,1.27)	0.83 (0.67,1.04)	0.84 (0.67,1.05)	0.69 (0.53,0.90)	0.81 (0.72,0.90)	<0.001
*Fiber from fruits*							
Case number	185	197	186	185	187		
Model 1	1 (ref)	1.00 (0.82, 1.23)	0.93 (0.76, 1.14)	0.90 (0.73, 1.11)	0.90 (0.74, 1.11)	0.99 (0.92, 1.06)	0.760
Model 2	1 (ref)	1.06 (0.87, 1.30)	1.00 (0.82, 1.24)	0.98 (0.79, 1.20)	0.94 (0.76, 1.17)	0.99 (0.92, 1.07)	0.822
*Fiber from vegetables*							
Case number	211	186	202	180	161		
Model 1	1 (ref)	0.87 (0.72, 1.07)	0.95 (0.78, 1.15)	0.85 (0.69, 1.03)	0.76 (0.62, 0.93)	0.89 (0.82, 0.97)	0.008
Model 2	1 (ref)	0.89 (0.73, 1.08)	0.94 (0.77, 1.14)	0.80 (0.65, 0.99)	0.65 (0.52, 0.83)	0.82 (0.74, 0.91)	<0.001
*Fiber from beans*							
Case number	198	197	203	153	189		
Model 1	1 (ref)	0.98 (0.81, 1.20)	1.03 (0.85, 1.25)	0.77 (0.62, 0.95)	0.96 (0.78, 1.17)	0.95 (0.88, 1.03)	0.214
Model 2	1 (ref)	1.01 (0.83, 1.23)	1.05 (0.86, 1.28)	0.77 (0.62, 0.96)	0.89 (0.71, 1.10)	0.90 (0.83, 0.99)	0.023
*Fiber from grains*							
Case number	196	205	169	198	172		
Model 1	1 (ref)	1.02 (0.84, 1.24)	0.83 (0.68, 1.02)	0.97 (0.79, 1.18)	0.82 (0.67, 1.01)	0.93 (0.86, 1.00)	0.055
Model 2	1 (ref)	1.02 (0.83, 1.24)	0.82 (0.67, 1.02)	0.95 (0.77, 1.17)	0.78 (0.62, 0.99)	0.91 (0.84, 0.99)	0.037

The SDs of intake were 0.86 oz/day for whole grains, 10.9 g/day for total dietary fiber, 3.6 g/day for fiber from fruits, 4.8 g/day for fiber from vegetables, 2.9 g/day for fiber from beans, and 4.0 g/day for fiber from grains.

Cox proportional hazard regression models were used. All *P*-values were two-sided. Model 1 were stratified by sex, adjusted for age at baseline (continuous). Model 2 further adjusted for level of education (‘≤11 years’, ‘high school’, ‘vocational technology school’, ‘some college’, ‘college/postgraduate’), race (‘non-Hispanic white’, ‘non-Hispanic black’, ‘Hispanic’, ‘Asian, Pacific Islander, or American Indian/Alaskan native’), BMI ('<25', '25–30', '30+' kg/m^2^), alcohol use ('non-drinker', '0.1–4.9', '5–9.9', '10+',gram/day), tobacco smoking ('never smoked', 'former smoker', 'current smoker'), physical activity ('never', 'rarely', '1–3 time per month', '1–2 times per week', '3–4 times per week', '5 + times per week'), history of diabetes ('no', 'yes') and total energy intake (continuous).

**Fig. 1:**
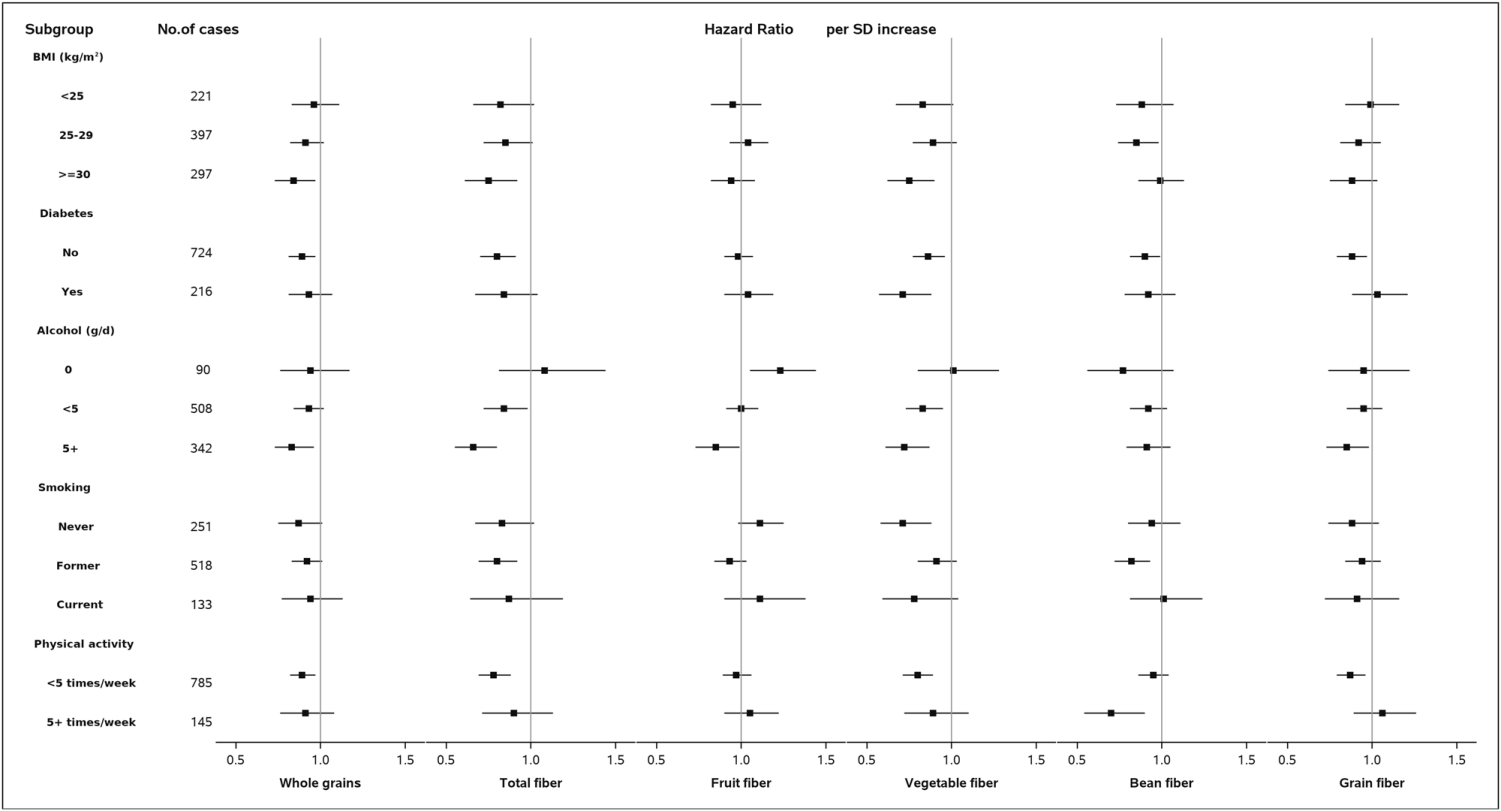
Stratified analyses for association between whole grain and dietary fiber intake with risk of liver cancer among the participants of NIH–AARP Diet and Health Study. Cox proportional hazard regression models were used. All *P*-values were two-sided. Models were stratified by sex and adjusted for age at baseline (continuous), level of education (‘≤11 years’, ‘high school’, ‘vocational technology school’, ‘some college’, ‘college/postgraduate’), race (‘non-Hispanic white’, ‘non-Hispanic black’, ‘Hispanic’, ‘Asian, Pacific Islander, or American Indian/Alaskan native’), BMI (‘ < 25’, ‘25–30’, ‘30 +’ kg/m^2^), alcohol use (‘Non-drinker’, ‘0.1–4.9’, ‘5–9.9’, ‘10 +’ g/day), tobacco smoking (‘never smoked’, ‘former smoker’, ‘current smoker’), physical activity (‘never’, ‘rarely’, ‘1–3 time per month’, ‘1–2 times per week’, ‘3–4 times per week’, ‘5+ times per week’), history of diabetes (‘no’, ‘yes’) and total energy intake (continuous), except when the variable is used for stratification. The *P* for interaction between whole grain or fiber intake and characteristics for stratification in risk of liver cancer were as follows: Whole grain: 0.22 for BMI, 0.25 for diabetes, 0.20 for alcohol use, 0.42 for smoking, and 0.98 for physical activity. Total fiber: 0.87 for BMI, 0.18 for diabetes, 0.02 for alcohol use, 0.76 for smoking, and 0.53 for physical activity. Fiber from fruits: 0.71 for BMI, 0.36 for diabetes, <0.01 for alcohol use, 0.41 for smoking, and 0.29 for physical activity. Fiber from vegetables: 0.68 for BMI, 0.54 for diabetes, 0.05 for alcohol use, 0.37 for smoking, and 0.57 for physical activity. Fiber from beans: 0.15 for BMI, 0.33 for diabetes, 0.35 for alcohol use, 0.63 for smoking, and 0.02 for physical activity. Fiber from grains: 0.69 for BMI, 0.02 for diabetes, 0.44 for alcohol use, 0.51 for smoking, and 0.23 for physical activity. Abbreviations: BMI body mass index; SD standard deviation; NIH–AARP: National Institutes of Health–American Association of Retired Persons.

In stratified analyses, the inverse associations of whole grains and risk of liver cancer were generally observed in different subgroups (Fig. 1).

### Whole grain, dietary fiber intake, and CLD mortality

A total of 993 CLD deaths were identified. Whole grain intake was associated with a 56% reduction of CLD mortality (HR_Q5 vs. Q1_ = 0.44, 95% CI: 0.35–0.55, *P*_*trend*_ < 0.001) after multivariable adjustment. Total dietary fiber intake was associated with a 63% reduction of CLD mortality (HR_Q5 vs. Q1_ = 0.37, 95% CI: 0.29–0.48, *P*_*trend*_ < 0.001) (Table 3). For different sources of fiber, the inverse associations were observed for vegetables (HR_Q5 vs. Q1_ = 0.55, 95% CI: 0.44–0.69, *P*_*trend*_ < 0.001), beans (HR_Q5 vs. Q1_ = 0.67, 95% CI: 0.54–0.84, *P*_*trend*_ = 0.03) and grains (HR_Q5 vs. Q1_ = 0.29, 95% CI: 0.23–0.36, *P*_*trend*_ < 0.001), but not for fruits (HR_Q5 vs. Q1_ = 0.98, 95% CI: 0.79–1.20, *P*_*trend*_ = 0.92). Similar results were observed in both men (Supplementary Table 7) and women (Supplementary Table 8).

**Table 3:** The associations between whole grains and dietary fiber intake with CLD mortality from the NIH–AARP Diet and Health Study.

	HR (95% CI)						*P trend*
	Quintile 1	Quintile 2	Quintile 3	Quintile 4	Quintile 5	Per SD increase	
*Whole grains*							
Case number	310	220	196	141	126		
Model 1	1 (ref)	0.69 (0.58, 0.82)	0.59 (0.49, 0.71)	0.43 (0.35, 0.53)	0.38 (0.31, 0.47)	0.67 (0.61, 0.73)	< 0.001
Model 2	1 (ref)	0.77 (0.65, 0.92)	0.69 (0.57, 0.82)	0.51 (0.42, 0.63)	0.44 (0.35, 0.55)	0.72 (0.66, 0.78)	< 0.001
*Total dietary fiber*							
Case number	305	214	197	130	147		
Model 1	1 (ref)	0.68 (0.57, 0.82)	0.62 (0.52, 0.74)	0.41 (0.33, 0.50)	0.46 (0.38, 0.56)	0.67 (0.61, 0.73)	<0.001
Model 2	1 (ref)	0.67 (0.56, 0.80)	0.59 (0.48, 0.71)	0.36 (0.29, 0.46)	0.37 (0.29, 0.48)	0.61 (0.54, 0.68)	<0.001
*Fiber from fruits*							
Case number	246	203	191	179	174		
Model 1	1 (ref)	0.78 (0.65, 0.94)	0.72 (0.60, 0.87)	0.66 (0.55, 0.80)	0.64 (0.52, 0.77)	0.86 (0.79, 0.93)	<0.001
Model 2	1 (ref)	0.95 (0.79, 1.15)	0.97 (0.80, 1.17)	0.95 (0.78, 1.17)	0.98 (0.79, 1.20)	1.00 (0.92, 1.07)	0.923
*Fiber from vegetables*							
Case number	250	218	207	169	149		
Model 1	1 (ref)	0.87 (0.72, 1.04)	0.82 (0.68, 0.98)	0.67 (0.55, 0.82)	0.59 (0.49, 0.73)	0.79 (0.72, 0.86)	<0.001
Model 2	1 (ref)	0.88 (0.73, 1.06)	0.82 (0.68, 0.99)	0.65 (0.53, 0.80)	0.55 (0.44, 0.69)	0.76 (0.69, 0.85)	<0.001
*Fiber from beans*							
Case number	223	230	209	175	156		
Model 1	1 (ref)	1.02 (0.85, 1.22)	0.94 (0.78, 1.13)	0.78 (0.64, 0.95)	0.70 (0.57, 0.86)	0.91 (0.83, 0.99)	0.025
Model 2	1 (ref)	1.03 (0.86, 1.24)	0.94 (0.77, 1.14)	0.77 (0.63, 0.94)	0.67 (0.54, 0.84)	0.90 (0.82, 0.99)	0.028
*Fiber from grains*							
Case number	328	230	186	127	122		
Model 1	1 (ref)	0.69 (0.58, 0.81)	0.55 (0.46, 0.66)	0.37 (0.30, 0.46)	0.35 (0.29, 0.43)	0.63 (0.58, 0.69)	<0.001
Model 2	1 (ref)	0.66 (0.55, 0.78)	0.50 (0.42, 0.61)	0.32 (0.26, 0.40)	0.29 (0.23, 0.36)	0.59 (0.53, 0.65)	<0.001

The SDs of intake were 0.86 oz/day for whole grains, 10.9 g/day for total dietary fiber, 3.6 g/day for fiber from fruits, 4.8 g/day for fiber from vegetables, 2.9 g/day for fiber from beans, and 4.0 g/day for fiber from grains.

Cox proportional hazard regression models were used. All *P*-values were two-sided. Model 1 were stratified by sex, adjusted for age at baseline (continuous). Model 2 further adjusted for level of education (‘≤11 years’, ‘high school’, ‘vocational technology school ’, ‘some college’, ‘college/postgraduate’), race (‘non-Hispanic white’, ‘non-Hispanic black’, ‘Hispanic’, ‘Asian, Pacific Islander, or American Indian/Alaskan native’), BMI ('<25', '25–30', '30+', kg/m^2^), alcohol use ('Non-drinker', '0.1–4.9', '5–9.9', '10+',gram/day), tobacco smoking ('never smoked', 'former smoker', 'current smoker'), physical activity ('never', 'rarely', '1–3 time per month', '1–2 times per week', '3–4 times per week', '5+ times per week'), history of diabetes ('no', 'yes') and total energy intake (continuous).

**Fig. 2:**
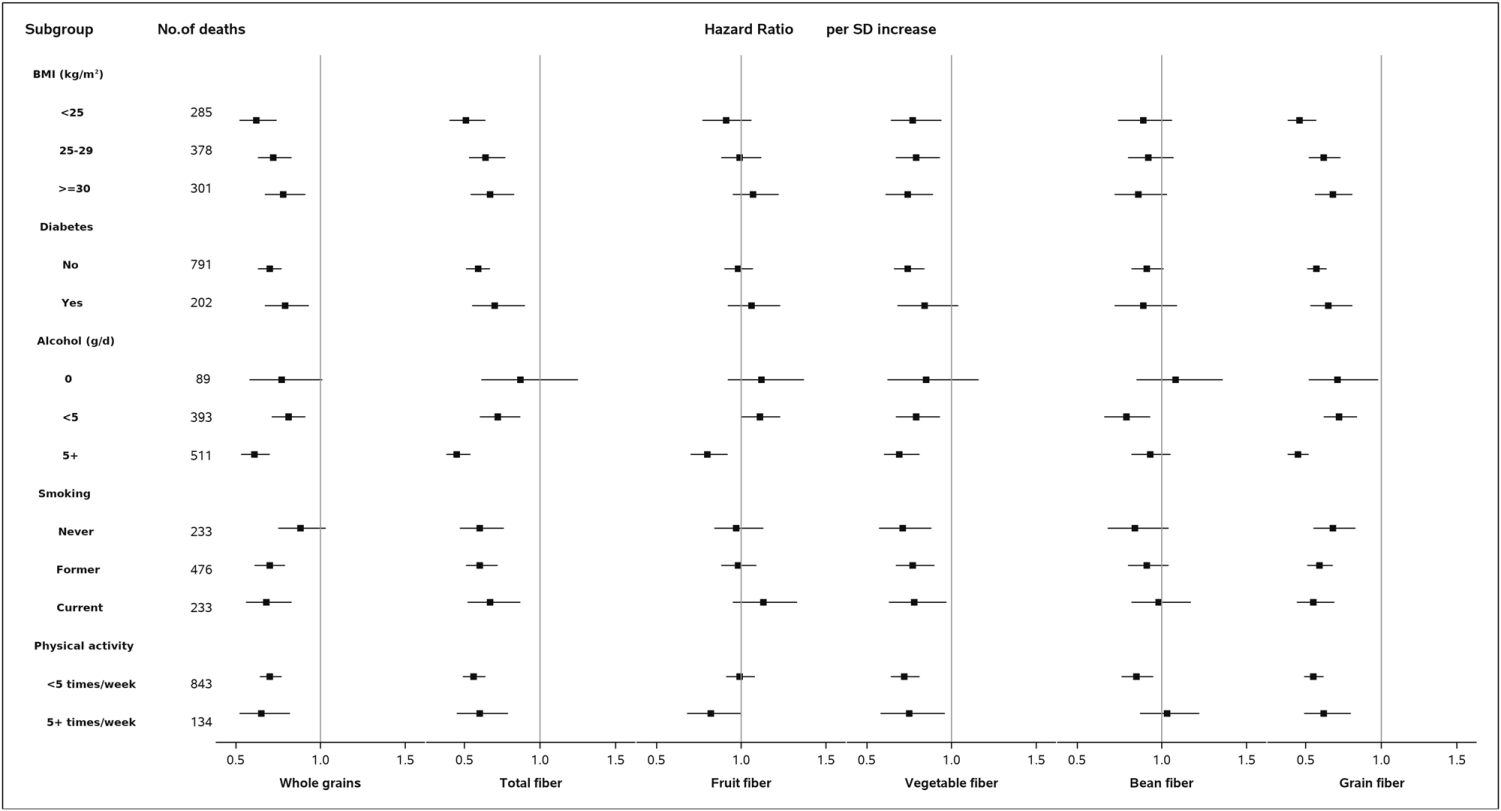
Stratified analyses for association between whole grain and dietary fiber intake with CLD mortality among the participants of NIH–AARP Diet and Health Study. Cox proportional hazard regression models were used. All *P*-values were two-sided. Models were stratified by sex and adjusted for age at baseline (continuous), level of education (‘≤11 years’, ‘high school’, ‘vocational technology school ’, ‘some college’, ‘college/postgraduate’), race (‘non-hispanic white’, ‘non-hispanic black’, ‘hispanic’, ‘asian, Pacific Islander, or American Indian/Alaskan native’), BMI (‘ < 25’, ‘25–30’, ‘30 +’ , kg/m^2^), alcohol use (‘non-drinker’, ‘0.1–4.9’, ‘5–9.9’, ‘10 + ’, gram/day), tobacco smoking (‘never smoked’, ‘former smoker’, ‘current smoker’), physical activity (‘never’, ‘rarely’, ‘1–3 time per month’, ‘1–2 times per week’, ‘3–4 times per week’, ‘5 + times per week’), history of diabetes (‘no’, ‘yes’) and total energy intake (continuous), except when the variable is used for stratification. The *P* for interaction between whole grain or fiber intake and characteristics for stratification in CLD mortality were as follows: Whole grain: 0.01 for BMI, 0.07 for diabetes, <0.01 for alcohol use, <0.01 for smoking, and 0.73 for physical activity. Total fiber: 0.03 for BMI, 0.05 for diabetes, <0.01 for alcohol use, 0.96 for smoking, and 0.52 for physical activity. Fiber from fruits: <0.01 for BMI, 0.10 for diabetes, <0.01 for alcohol use, 0.97 for smoking, and 0.16 for physical activity. Fiber from vegetables: 0.83 for BMI, 0.11 for diabetes, 0.72 for alcohol use, 0.63 for smoking, and 0.76 for physical activity. Fiber from beans: 0.87 for BMI, 0.84 for diabetes, 0.23 for alcohol use, 0.07 for smoking, and 0.07 for physical activity. Fiber from grains: <0.01 for BMI, 0.13 for diabetes, <0.01 for alcohol use, 0.15 for smoking, and 0.40 for physical activity. Abbreviations: BMI body mass index; SD standard deviation; NIH–AARP National Institutes of Health–American Association of Retired Persons.

In stratified analyses, the significant inverse associations of whole grain, dietary fiber, and CLD mortality were observed across most subgroups (Fig. 2).

### Sensitivity analyses observed similar results

In sensitivity analyses, most of the inverse associations for risk of liver cancer and CLD mortality remained essentially unchanged when we further adjusted for HEI-2015, or excluded cases diagnosed within the first 2 or 5 years of follow-up, or excluded heavy alcohol drinkers.

## Discussion

In this cohort study, participants with the highest quintile of whole grain intake had a 22% reduced risk of liver cancer and a 56% reduction in CLD mortality. Participants with the highest quintile of total dietary fiber intake had a 31% reduced risk of liver cancer and a 63% reduction in CLD mortality. These inverse associations were observed for fiber from vegetables, beans, and grains but not from fruits.

A recent systematic review reported an inverse association between higher whole grain intake and HCC risk^23^. Thus far, two cohort studies have reported associations between dietary fiber intake and risk of liver cancer. The European Prospective Investigation into Cancer and Nutrition (EPIC) (*n* = 191 HCC cases) reported significant associations for total fiber (HR_Q4 vs. Q1_ = 0.51, 95% CI: 0.31–0.83) and fiber from cereals, but not from vegetables, fruits, and other sources^24^. The Nurses’ Health Study (NHS) and the Health Professionals Follow-up Study (HPFS) (*n* = 141 HCC cases) reported a suggestive association between fiber from cereals (HR_T3 vs. T1_ = 0.68, 95% CI: 0.45–1.03, *P*_trend_ = 0.07), but not fiber from fruits or vegetables and HCC risk^25^. Moreover, the NHS/HPFS study also found an inverse association between whole grain intake and HCC risk (HR_T3 vs. T1_ = 0.63, 95% CI: 0.41 to 0.96)^25^. In this current study, no association was found for fiber from fruits, and an inverse association was found for whole grains and total fiber, which were consistent with the observation from the EPIC^24^ and NHS/HPFS studies^25^. Moreover, inverse associations were observed for fiber from vegetables and grains in this study, adding to the knowledge that fiber from different food sources might have different associations with liver cancer. Furthermore, this study reports the inverse associations between whole grains, total dietary fibers, fibers from vegetables, and grains with CLD mortality, expanding the potential benefit of whole grain and dietary fiber intake from the risk of liver cancer to a wide range of chronic liver diseases.

The biologic mechanisms for the inverse associations of whole grains and dietary fiber with the liver disease remain to be fully elucidated. Whole grains are good sources of dietary fiber, resistant starch, and oligosaccharides, which may increase stool bulk, speed intestinal transit and reduce the exposure to carcinogens in the colon^35^. Whole grains contain antioxidants and phytoestrogens and have been reported to have a protective effect among certain gastrointestinal cancers and hormone-sensitive cancers^36^. Dietary fiber has been reported to have beneficial effects on conditions related to liver disease and liver cancer, including blood glucose^37^, insulin sensitivity^37^, liver fat content^38^, and metabolic syndrome^39–41^. Oligosaccharides as fermentable carbohydrates are fermented in the colon and increase the production of short-chain fatty acids, which are important in the maintenance of gut integrity and regulation of inflammation^36,42^. Recent studies discovered that the effects of dietary fiber might be mediated by the alteration in gut microbiota, such as an increased abundance of *Prevotella, Treponema,* and *Succinivibrio*^43–45^. Moreover, increasing evidence revealed the existence of a gut–liver axis, which exchanges signals between the bowel and liver. The portal vein allows transporting products generated by microbial communities to the liver, and the microbial communities are critical in the homeostasis of the gut–liver axis. Given that evidence accumulates for the pathogenetic role of microbe-derived metabolites in NAFLD, a similar mechanism may apply to more advanced liver diseases^46^. Our results also found heterogeneous associations for fiber from different food sources. Beans, vegetables, and fruits are good sources of dietary fiber. Interestingly, fiber from fruits did not show an inverse association with liver disease mortality, as did fiber from other sources. Although the underlying mechanism was unclear, excess fructose has been reported to be associated with increased insulin resistance, fatty acid production, oxidative stress, and fatty liver^47^. Fruits high in fructose might cancel out the beneficial effect of fiber due to an adverse effect of fructose^48^. Collectively, our study suggests that the inverse association between dietary fiber and liver disease mortality may vary depending on the source of fiber. More investigation is needed to further clarify the potential mechanisms.

The present study is the largest cohort study to date to evaluate the associations between whole grain and dietary fiber intake with liver cancer risk. In addition, it reports an association between dietary fiber intake and CLD mortality. With the large sample size, dietary fiber from different food sources was examined in detail, and two major subtypes of primary liver cancer (i.e., HCC and ICC) were studied. Many of the important lifestyle confounders for liver cancer and CLD, including age, alcohol, tobacco use, obesity, and diabetes, were included in the analyses. However, there are several limitations to this study. First, as important risk factors for liver cancer and CLD, HBV/HCV was not measured in the full cohort, neither was information on history of hepatitis collected. It is estimated that the prevalence is 0.1% for HBV and 1.0% for HCV in the US population^49,50^. Using the data from the Liver Cancer Pooling Project and NHS/HPFS cohorts, we and others have reported no correlations between HBV/HCV infections with BMI^51^, smoking^52^, alcohol use^52^, coffee intake^53^, fiber^25^, nuts^54^, fatty acids^55^, dairy foods^56^, or meats^57^. Furthermore, previous studies have reported similar findings with or without adjustment for HBV/HCV for associations of liver cancer risk with tooth loss^58^, fish intake^59^, and coffee^60^. Thus, the relationship between diet and liver cancer risk in this study is unlikely to be substantially confounded by HBV/HCV infection status. Second, whole grain and dietary fiber intake were derived from the self-reported FFQ. The FFQ was validated by using two non-consecutive 24-h dietary recalls. The energy-adjusted correlation coefficients for dietary fiber intake were 0.72 among men and 0.66 among women^31^. Measurement errors cannot be ruled out. We examined the associations for dietary fiber and whole grain based on ranking instead of absolute intake levels, because the FFQ is better suited to ranking according to relative intake than assessing precise intake quantitatively. Third, the food intake questionnaire was self-administered at baseline. Thus, the change of diet during the follow-up was not recorded or analyzed. We cannot evaluate the associations between changes of whole grain and dietary fiber intake with the outcomes. Fourth, people who had higher whole grain or fiber intake were more likely to have a healthier diet and lifestyle overall, which might contribute to the reduced risk. Residual confounding might exist. Fifth, in this analysis, the outcomes of interest were liver cancer incidence and CLD mortality. While it would have been ideal for examining CLD incidence in addition to morality, CLD registries do not exist in the US, nor is there a single medical records system to permit the identification of CLD diagnoses. As a result, CLD mortality was analyzed in the current study. Finally, the study participants are predominantly persons of European ancestry and are individuals aged 50–71 years old at the beginning of the follow-up, which potentially limited the generalizability of the results to other populations.

In conclusion, we found significant inverse associations between whole grain and dietary fiber intake with liver cancer incidence and CLD mortality in a US population. The associations between dietary fiber were particular for fiber from vegetables, beans, and grains. These findings, if confirmed by future studies, indicate that a diet rich in whole grain and dietary fiber could be recommended for reducing liver cancer incidence and CLD mortality. Further studies are warranted to clarify the underlying mechanisms and determine specific dietary components associated with benefit in diverse racial/ethnic populations.

## Methods

### Study population

The NIH–AARP Diet and Health Study recruited men and women aged 50-71 years old by mailing questionnaires to its 3.5 million members from six US states (California, Florida, Louisiana, New Jersey, North Carolina, and Pennsylvania) and two metropolitan areas (Atlanta, GA and Detroit, MI) in 1995–1996^26^. A total of 566,398 persons (339,666 men and 226,732 women) returned the baseline questionnaire and were included in the final cohort. In this analysis, we excluded participants with: (1) prevalent cancer (*n* = 50,118) at baseline; (2) extreme caloric intakes (<500, or >3,500 for women; <800, or >4,000 for men; *n* = 29,983); and (3) participants who were diagnosed with liver cancer or died from CLD before their baseline survey questionnaires were scanned (*n* = 580). Thus, the analytic cohort included 485,717 (290,484 men and 195,233 women). The study was approved by the Special Studies Institutional Review Board of the U.S. National Cancer Institute. Informed consent has been obtained from all participants.

### Case ascertainment

#### Liver cancer

Cases of primary liver cancer (defined by International Classification of Diseases, 10th edition [ICD-10] diagnostic code C22) were identified by linkage with state cancer registries through December 31^st^, 2011^26^. Cases of HCC were further defined by morphology codes 8170–8175. Cases of ICC were defined by morphology codes 8032, 8033, 8070, 8071, 8140, 8141, 8160, 8161, 8260, 8480, 8481, 8490 and 8560^27^. Prior examination reported that these codes have a sensitivity of 90% and a specificity of nearly 100% for cancer identification^28^.

#### Deaths due to chronic liver diseases

The National Death Index Plus (NDIP) was used to identify causes of death through December 31^st^, 2011. Consistent with previous studies in the same cohort^28,29^, deaths due to chronic liver diseases included deaths from fibrosis, cirrhosis, alcoholic liver diseases, and chronic hepatitis (ICD-9: 571.0, 571.2–571.6, 571.8, and 571.9; ICD-10: K70, K73, and K74). Classification of CLD from the NDIP was validated using the electronic medical records from members of the Kaiser Permanente Medical Care Program of Northern California, and specificity was found to be 89%^30^.

### Dietary assessment

The information on diet was collected via a 124-item food-frequency questionnaire (FFQ)^26^. The FFQ was validated among 2,000 NIH–AARP participants using two 24-h dietary recalls and a second FFQ^26,31^. The energy-adjusted correlation coefficients for dietary fiber intake were 0.72 in men and 0.66 in women^31^. Food groups were created with the MyPyramid Equivalents Database (MPED) version 1.0, and component ingredients were disaggregated from food mixtures, assigned to food groups, and calculated to cup or ounce equivalents^32^. Nutrients were calculated with the 1994–1996 U.S. Department of Agriculture’s Continuing Survey of Food Intakes by Individuals (CSFII)^33^. The assessments of whole grain and fiber intake were described in the previous study from the same cohort^34^. Specifically, whole grains were defined and extracted as the whole grain part of each food item, including ready-to-eat cereals, high-fiber cereals, cooked cereals, other fiber cereals, whole-grain bread or dinner rolls, popcorn, pancakes, waffles, French toast or crepes, rice, or other cooked grains, bagels, English muffins, tortillas, pasta, crackers, chips, cookies, or brownies, sweet pastries, and pies. Whole grain intake was presented as MPED number of whole grains in ounce equivalents per day (oz eq/day). The USDA’s Pyramid Servings Database was used to estimate whole grain intake from all foods collected in the FFQ, even from those foods with small amounts of whole grain. Total fiber from fruits, vegetables, beans and grains was estimated by summing dietary fiber from these foods. In the questionnaire, the participants were asked about the frequency and amount of intake for different kinds of vegetables and fruits. They were also asked about beans including ‘beans, such as baked beans, refried beans, pintos, kidney, lima, lentils, or soybeans (not including bean soup)’ and ‘bean-based soups’. For fruits and vegetables, a 1-cup equivalent is defined as 1-cup raw or cooked fruits or vegetables, 1-cup fruit or vegetable juice, 0.5 cup dried fruits, or 2-cups leafy greens. For beans, a 1-cup equivalent is defined as 1-cup cooked dry beans or peas. For grains, a 1-ounce equivalent is defined as one regular slice of yeast bread, or 0.5-cup rice, pasta, or cooked cereal, or 1 cup ready-to-eat cereal. The equivalents were further converted into gram amounts^32^.

### Covariate assessment

Socio-demographic characteristics including age, race (self-reported), weight, height, level of education, and other potential risk factors including tobacco smoking, alcohol drinking, physical activity, self-reported history of diabetes were also collected in the baseline questionnaire^26^. Participants reported their highest level of education from the following categories: <8 years; 8–11 years; 12 years, or completed high school; post-high school training other than college (e.g., vocational or technical training); some college; college graduate; postgraduate. The frequency and amount of reported alcohol consumption (including beer, wine, or wine coolers, and liquor or mixed drinks) were converted to grams per day in analyses. Participants reported whether they had ever smoked 100 or more cigarettes during lifetime and if they currently smoked or had stopped. Frequency (never, rarely, 1–3 times per month, 1–2 times per week, 3–4 times per week, 5 + times per week) in the past 12 months of physical activity at work or home (including exercise, sports, and activities such as carrying heavy loads) ≥ 20 minutes that caused increases in breathing or heart rate or sufficient to work up a sweat was asked.

### Statistical analyses

For liver cancer, person-years to the event was calculated for each participant from baseline to date of diagnosis of primary liver cancer, date of death, or end of follow-up (December 31^st^, 2011), whichever came the first. For CLD mortality, person-years to the event was calculated from baseline to date of CLD death, date of death from other reasons, or end of follow-up (December 31^st^, 2011), whichever came the first. Cox proportional hazard regression models were used to estimate hazard ratios (HRs) and 95% confidence intervals (CIs) for events comparing across quintile categories of whole grain or dietary fiber intake with the lowest intake quintile as the reference group. The proportional hazards assumption was tested using a cross-product term of log(time) and whole grain or dietary fiber intake; no violations were observed (*P* > 0.05*)*. *P* for trend was calculated per SD increase in whole grain or dietary fiber intake. The associations of whole grain and fiber intake with liver cancer incidence and CLD mortality were not modified by sex (*P* for heterogeneity > 0.05). Therefore, we report pooled estimates, with sex-specific results presented in Supplementary Tables.

Multivariable models included the following a priori covariates: age at baseline (continuous), level of education (≤ 11 years; high school; vocational technology school; some college; college/postgraduate), race (non-Hispanic white; non-Hispanic black; Hispanic; Asian, Pacific Islander, or American Indian/Alaskan Native), body mass index (BMI, < 25; 25–30; ≥30 kg/m^2^), alcohol consumption (non-drinker; 0.1–4.9; 5–9.9; ≥10 g/day), tobacco smoking (never smoked; former smoker; current smoker), physical activity (never; rarely; 1–3 time per month; 1–2 times per week; 3–4 times per week; 5 or more times per week), self-reported history of diabetes (no; yes), and total energy intake (continuous).

Subgroup analyses were conducted across categories of BMI, self-reported history of diabetes, alcohol drinking, tobacco smoking, and physical activity. *P* for interaction was tested by introducing a product term of the two variables examined in the regression models.

In sensitivity analyses, the healthy eating index (HEI-2015) was added to the multivariable regression models to control for overall dietary quality. Second, we excluded cases diagnosed within the first 2 or 5 years of follow-up to address the possible reverse causation. We also excluded heavy alcohol drinkers, defined as men who consumed more than 4 drinks and women who consumed more than 3 drinks per day, to further control for confounding from alcohol drinking.

SAS 9.4 software was used for all analyses (SAS Institute Inc., Cary, NC, USA). All *P*-values were two-sided.

### Reporting Summary

Further information on research design is available in the Nature Research Reporting Summary linked to this article.

## Supplementary information

Supplementary Information

Reporting Summary

## Data Availability

Data used in this study are maintained by the National Cancer Institute, Division of Cancer Epidemiology and Genetics, and are available upon submitting a proposal to be approved by the NIH–AARP Steering Committee for privacy concerns. For more information, visit https://www.nihaarpstars.com/.

## Code availability statement

Code used in this study is available; please contact the corresponding author.
