# Supplementary material for: Higher intake of whole grains and dietary fiber are associated with lower risk of liver cancer and chronic liver disease mortality
Source: Nat Commun. 2021 Nov 4;12:6388. doi: 10.1038/s41467-021-26448-9 (PMC8568891; doi:10.1038/s41467-021-26448-9)
Supplement: Supplementary file 1 — Supplementary Information [file 41467_2021_26448_MOESM1_ESM.pdf]

Supplementary Table 1 Baseline characteristics according to whole grains and total fiber intake among male participants of NIH-AARP Diet and Health Study

	Quintile categories for whole grains intake, oz/day					Quintile categories for total fiber intake, gram/day				
	'<=0.39' (n=57757)	'>0.39-<=0.71' (n=57724)	'>0.71-<=1.08' (n=59513)	'>1.08-<=1.59' (n=57289)	'>1.59' (n=58201)	'<=12.57' (n=58086)	'>12.57-<=16.44' (n=58120)	'>16.44-<=20.46' (n=58073)	'>20.46-<=26.16' (n=58132)	'>26.16' (n=58073)
Age, yr	61.6 (5.4)	61.6 (5.4)	61.6 (5.4)	61.6 (5.4)	61.6 (5.4)	61.6 (5.4)	61.6 (5.4)	61.6 (5.4)	61.6 (5.4)	61.6 (5.4)
Female, %	0.0 (0.0)	0.0 (0.0)	0.0 (0.0)	0.0 (0.0)	0.0 (0.0)	0.0 (0.0)	0.0 (0.0)	0.0 (0.0)	0.0 (0.0)	0.0 (0.0)
White, %	92.7	93.4	94.6	94.8	94.0	92.9	94.5	94.8	94.6	92.8
College and above, %	38.4	44.9	47.7	49.7	47.5	41.1	44.7	46.5	47.6	48.3
BMI, kg/m^2	27.4 (4.4)	27.5 (4.3)	27.3 (4.2)	27.1 (4.2)	26.9 (4.3)	27.3 (4.2)	27.3 (4.2)	27.3 (4.2)	27.2 (4.3)	27.1 (4.5)
Physical activity 5+ times/week, %	16.7	19.0	20.9	23.3	27.1	13.8	17.7	20.8	23.8	31.0
Alcohol, gram/day	19.5 (37.2)	15.6 (29.8)	14.2 (26.7)	13.2 (24.9)	10.8 (21.2)	16.4 (32.7)	15.4 (30.1)	15.3 (29.2)	14.2 (27.0)	12.0 (22.9)
Current smoking, %	17.5	11.4	9.2	7.6	7.5	16.5	12.0	9.8	8.5	6.4
Self-reported diabetes, %	8.5	9.6	9.9	10.4	12.2	8.7	9.7	10.1	10.5	11.6
Whole grains, oz/d	0.2 (0.1)	0.6 (0.1)	0.9 (0.1)	1.3 (0.1)	2.4 (0.9)	0.5 (0.4)	0.8 (0.5)	1.0 (0.6)	1.3 (0.8)	1.8 (1.2)
Dietary fiber intake, types, and food sources, g/d										
Total	14.1 (6.8)	16.9 (7.0)	19.1 (7.2)	21.9 (7.8)	27.6 (9.7)	9.8 (2.1)	14.5 (1.1)	18.4 (1.2)	23.0 (1.6)	33.8 (7.8)
Fruits	3.0 (3.0)	3.6 (3.0)	4.0 (3.1)	4.5 (3.3)	5.2 (3.8)	1.7 (1.2)	2.8 (1.7)	3.7 (2.1)	4.8 (2.6)	7.4 (4.6)
Vegetables	5.2 (3.4)	5.9 (3.4)	6.3 (3.5)	6.9 (3.8)	8.0 (4.6)	3.2 (1.4)	4.8 (1.7)	6.0 (2.1)	7.4 (2.6)	11.0 (5.0)
Beans	1.9 (2.1)	2.2 (2.3)	2.3 (2.3)	2.6 (2.6)	3.1 (3.2)	1.1 (0.8)	1.6 (1.2)	2.1 (1.5)	2.7 (2.0)	4.6 (4.2)
Grains	3.7 (2.4)	5.0 (2.4)	6.2 (2.4)	7.7 (2.6)	11.0 (3.9)	3.6 (1.5)	5.2 (1.9)	6.5 (2.3)	7.9 (2.9)	10.6 (4.7)

Values are means (SD) for continuous variables, percentages for categorical variables, and are standardized to the age distribution of the study population.

[*] Value is not age adjusted.

Supplementary Table 2 Baseline characteristics according to whole grains and total fiber intake among female participants of NIH-AARP Diet and Health Study

	Quintile categories for whole grain intake, oz/day					Quintile categories for total fiber intake, gram/day				
	'<=0.32' (n=38924)	'>0.32-<=0.57' (n=38521)	'>0.57-<=0.87' (n=39729)	'>0.87-<=1.29' (n=38861)	'>1.29' (n=39198)	'<=10.65' (n=39093)	'>10.65-<=14.25' (n=39019)	'>14.25-<=17.99' (n=39022)	'>17.99-<=23.37' (n=39057)	'>23.37' (n=39042)
Age, yr	61.4 (5.4)	61.4 (5.4)	61.4 (5.4)	61.4 (5.4)	61.4 (5.4)	61.4 (5.4)	61.4 (5.4)	61.4 (5.4)	61.4 (5.4)	61.4 (5.4)
Female, %	1.0 (0.0)	1.0 (0.0)	1.0 (0.0)	1.0 (0.0)	1.0 (0.0)	1.0 (0.0)	1.0 (0.0)	1.0 (0.0)	1.0 (0.0)	1.0 (0.0)
White, %	90.7	91.4	91.6	91.8	89.5	91.0	92.5	92.2	91.6	87.7
College and above, %	26.8	30.3	31.8	33.2	32.9	25.5	29.6	32.2	33.5	34.2
BMI, kg/m^2	26.7 (6.1)	26.9 (5.9)	26.9 (5.8)	26.8 (5.9)	26.8 (6.3)	26.7 (5.9)	26.9 (5.9)	26.8 (5.9)	26.9 (5.9)	26.8 (6.2)
Physical activity 5+time/week, %	13.6	15.1	16.0	17.2	19.8	10.6	13.5	15.8	18.2	23.7
Alcohol, gram/day	7.6 (19.5)	6.1 (15.4)	5.5 (12.9)	5.1 (12.2)	4.1 (10.4)	7.0 (18.3)	6.2 (15.6)	5.7 (14.0)	5.2 (12.5)	4.2 (10.4)
Current smoking, %	21.7	15.3	13.3	12.1	10.5	23.2	16.1	13.0	11.3	9.2
Self-reported diabetes, %	5.9	6.7	7.1	7.4	9.5	6.5	6.7	7.4	7.6	8.5
Whole grains, oz/d	0.2 (0.1)	0.5 (0.1)	0.7 (0.1)	1.1 (0.1)	2.0 (0.7)	0.4 (0.3)	0.6 (0.4)	0.8 (0.5)	1.1 (0.6)	1.5 (1.0)
Dietary fiber intake, types, and food sources, g/d										
Total	11.9 (6.5)	14.7 (6.5)	17.0 (6.9)	19.4 (7.3)	24.6 (9.1)	8.1 (1.9)	12.5 (1.0)	16.1 (1.1)	20.4 (1.5)	30.6 (7.4)
Fruits	3.1 (3.0)	3.7 (2.9)	4.1 (3.0)	4.5 (3.2)	5.3 (3.7)	1.7 (1.2)	2.8 (1.6)	3.8 (2.0)	5.0 (2.5)	7.6 (4.3)
Vegetables	4.9 (3.6)	5.6 (3.5)	6.1 (3.7)	6.6 (4.0)	7.7 (4.7)	2.9 (1.3)	4.4 (1.6)	5.5 (2.0)	7.1 (2.5)	11.1 (5.2)
Beans	1.2 (1.6)	1.5 (1.7)	1.6 (1.9)	1.8 (2.1)	2.3 (2.7)	0.7 (0.6)	1.1 (0.9)	1.4 (1.2)	1.9 (1.6)	3.3 (3.5)
Grains	2.6 (1.8)	3.8 (1.8)	4.9 (1.9)	6.2 (2.0)	9.1 (3.2)	2.7 (1.3)	4.1 (1.6)	5.1 (2.0)	6.3 (2.6)	8.4 (3.9)

Values are means (SD) for continuous variables, percentages for categorical variables, and are standardized to the age distribution of the study population.

* Value is not age adjusted.

Supplementary Table 3 The association between whole grains and dietary fiber with risk of liver cancer among male participants of NIH-AARP Diet and Health Study

	HR (95% CI)						*P trend*
	Quintile 1	Quintile 2	Quintile 3	Quintile 4	Quintile 5	Per SD increase	
Whole grains							
Case number	164	157	157	116	137		
Model 1	1 (ref)	0.93 (0.74, 1.15)	0.89 (0.71, 1.10)	0.67 (0.53, 0.85)	0.77 (0.62, 0.97)	0.90 (0.83, 0.97)	0.009
Model 2	1 (ref)	0.96 (0.77, 1.19)	0.96 (0.77, 1.19)	0.72 (0.57, 0.92)	0.80 (0.63, 1.02)	0.90 (0.83, 0.98)	0.012
Total dietary fiber							
Case number	149	171	133	141	137		
Model 1	1 (ref)	1.12 (0.90, 1.39)	0.86 (0.68, 1.08)	0.90 (0.71, 1.13)	0.87 (0.69, 1.09)	0.90 (0.82, 0.99)	0.032
Model 2	1 (ref)	1.09 (0.87, 1.36)	0.80 (0.63, 1.03)	0.81 (0.62, 1.05)	0.71 (0.53, 0.95)	0.81 (0.72, 0.92)	0.001
Fiber from fruits							
Case number	142	154	152	137	146		
Model 1	1 (ref)	1.03 (0.82, 1.29)	0.99 (0.79, 1.25)	0.87 (0.69, 1.11)	0.93 (0.73, 1.17)	0.98 (0.90, 1.06)	0.602
Model 2	1 (ref)	1.08 (0.86, 1.36)	1.08 (0.85, 1.36)	0.95 (0.74, 1.21)	0.98 (0.77, 1.25)	0.99 (0.91, 1.07)	0.746
Fiber from vegetables							
Case number	164	150	159	130	128		
Model 1	1 (ref)	0.91 (0.73, 1.13)	0.96 (0.77, 1.19)	0.79 (0.62, 0.99)	0.78 (0.62, 0.98)	0.89 (0.80, 0.98)	0.016
Model 2	1 (ref)	0.92 (0.73, 1.15)	0.94 (0.75, 1.18)	0.73 (0.57, 0.93)	0.65 (0.50, 0.85)	0.81 (0.73, 0.91)	<0.001
Fiber from beans							
Case number	144	151	166	119	151		
Model 1	1 (ref)	1.03 (0.82, 1.30)	1.16 (0.92, 1.44)	0.82 (0.64, 1.05)	1.04 (0.83, 1.31)	0.95 (0.87, 1.04)	0.294
Model 2	1 (ref)	1.05 (0.84, 1.33)	1.16 (0.93, 1.46)	0.81 (0.63, 1.04)	0.95 (0.74, 1.22)	0.90 (0.82, 0.99)	0.033
Fiber from grains							
Case number	151	164	125	155	136		
Model 1	1 (ref)	1.06 (0.85, 1.32)	0.80 (0.63, 1.01)	0.98 (0.78, 1.23)	0.84 (0.67, 1.07)	0.94 (0.87, 1.02)	0.150
Model 2	1 (ref)	1.05 (0.84, 1.32)	0.79 (0.62, 1.01)	0.97 (0.76, 1.23)	0.82 (0.63, 1.07)	0.93 (0.85, 1.03)	0.152

Cox proportional hazard regression models were used. All P-values were two-sided. Model 1 were adjusted for age at baseline (continuous). Model 2 further adjusted for level of education ('<=11 yrs', 'High school', 'Vocational technology school', 'Some College', 'College/Post Graduate'), race ('Non-Hispanic White', 'Non-Hispanic Black', 'Hispanic', 'Asian, Pacific Islander, or American Indian/Alaskan Native'), BMI ('<25', '25-<30', '30+', kg/m^2), alcohol use ('Non-drinker', '0.1-4.9', '5-9.9', '10+',gram/day), tobacco smoking ('Never smoked', 'Former smoker', 'Current smoker'), physical activity ('Never', 'Rarely', '1-3 time per month', '1-2 times per week', '3-4 times per week', '5+ times per week'), history of diabetes ('No', 'Yes') and total energy intake (continuous).

Supplementary Table 4 The association between whole grains and dietary fiber with risk of liver cancer among female participants of NIH-AARP Diet and Health Study

	HR (95% CI)						*P trend*
	Quintile 1	Quintile 2	Quintile 3	Quintile 4	Quintile 5	Per SD increase	
Whole grains							
Case number	50	37	41	39	42		
Model 1	1 (ref)	0.73 (0.48, 1.11)	0.77 (0.51, 1.17)	0.74 (0.49, 1.12)	0.78 (0.51, 1.17)	0.94 (0.79, 1.11)	0.449
Model 2	1 (ref)	0.74 (0.48, 1.14)	0.79 (0.52, 1.20)	0.75 (0.49, 1.16)	0.73 (0.47, 1.15)	0.90 (0.75, 1.09)	0.287
Total dietary fiber							
Case number	44	39	44	46	36		
Model 1	1 (ref)	0.86 (0.56, 1.32)	0.96 (0.63, 1.46)	0.99 (0.65, 1.50)	0.77 (0.50, 1.20)	0.90 (0.75, 1.08)	0.271
Model 2	1 (ref)	0.87 (0.56, 1.35)	0.96 (0.61, 1.50)	0.95 (0.59, 1.54)	0.66 (0.37, 1.18)	0.80 (0.62, 1.03)	0.085
Fiber from fruits							
Case number	43	43	34	48	41		
Model 1	1 (ref)	0.93 (0.61, 1.43)	0.72 (0.46, 1.13)	0.99 (0.66, 1.50)	0.83 (0.54, 1.28)	1.03 (0.89, 1.19)	0.729
Model 2	1 (ref)	0.99 (0.65, 1.52)	0.78 (0.49, 1.24)	1.07 (0.69, 1.64)	0.84 (0.52, 1.34)	1.02 (0.87, 1.19)	0.852
Fiber from vegetables							
Case number	47	36	43	50	33		
Model 1	1 (ref)	0.76 (0.49, 1.17)	0.90 (0.59, 1.36)	1.05 (0.71, 1.57)	0.70 (0.45, 1.10)	0.90 (0.75, 1.08)	0.257
Model 2	1 (ref)	0.79 (0.51, 1.22)	0.93 (0.60, 1.43)	1.06 (0.69, 1.63)	0.65 (0.39, 1.08)	0.85 (0.69, 1.04)	0.120
Fiber from beans							
Case number	54	46	37	34	38		
Model 1	1 (ref)	0.84 (0.57, 1.25)	0.69 (0.45, 1.05)	0.64 (0.41, 0.98)	0.72 (0.47, 1.09)	0.92 (0.74, 1.15)	0.478
Model 2	1 (ref)	0.90 (0.61, 1.34)	0.75 (0.49, 1.15)	0.67 (0.43, 1.05)	0.73 (0.46, 1.15)	0.92 (0.73, 1.15)	0.460
Fiber from grains							
Case number	45	41	44	43	36		
Model 1	1 (ref)	0.90 (0.59, 1.37)	0.95 (0.63, 1.44)	0.92 (0.60, 1.39)	0.76 (0.49, 1.17)	0.87 (0.72, 1.05)	0.138
Model 2	1 (ref)	0.90 (0.59, 1.39)	0.95 (0.61, 1.47)	0.90 (0.57, 1.43)	0.71 (0.42, 1.18)	0.83 (0.67, 1.03)	0.095

Cox proportional hazard regression models were used. All P-values were two-sided. Model 1 were adjusted for age at baseline (continuous). Model 2 further adjusted for level of education ('<=11 yrs', 'High school', 'Vocational technology school', 'Some College', 'College/Post Graduate'), race ('Non-Hispanic White', 'Non-Hispanic Black', 'Hispanic', 'Asian, Pacific Islander, or American Indian/Alaskan Native'), BMI ('<25', '25-<30', '30+', kg/m^2), alcohol use ('Non-drinker', '0.1-4.9', '5-9.9', '10+',gram/day), tobacco smoking ('Never smoked', 'Former smoker', 'Current smoker'), physical activity ('Never', 'Rarely', '1-3 time per month', '1-2 times per week', '3-4 times per week', '5+ times per week'), history of diabetes ('No', 'Yes') and total energy intake (continuous).

Supplementary Table 5 The association between whole grains and dietary fiber with risk of HCC among participants of NIH-AARP Diet and Health Study

	HR (95% CI)						*P trend*
	Quintile 1	Quintile 2	Quintile 3	Quintile 4	Quintile 5	Per SD increase	
Whole grains							
Case number	140	132	131	107	125		
Model 1	1 (ref)	0.91 (0.72, 1.16)	0.87 (0.68, 1.10)	0.72 (0.56, 0.93)	0.83 (0.65, 1.05)	0.92 (0.85, 1.00)	0.055
Model 2	1 (ref)	0.94 (0.74, 1.19)	0.93 (0.73, 1.18)	0.77 (0.59, 0.99)	0.81 (0.63, 1.04)	0.90 (0.83, 0.98)	0.020
Total dietary fiber							
Case number	125	144	118	136	112		
Model 1	1 (ref)	1.12 (0.88, 1.42)	0.90 (0.70, 1.16)	1.03 (0.81, 1.31)	0.84 (0.65, 1.09)	0.90 (0.82, 1.00)	0.049
Model 2	1 (ref)	1.08 (0.84, 1.38)	0.83 (0.64, 1.09)	0.89 (0.67, 1.17)	0.62 (0.45, 0.86)	0.76 (0.67, 0.87)	<0.001
Fiber from fruits							
Case number	117	131	133	124	130		
Model 1	1 (ref)	1.05 (0.82, 1.35)	1.05 (0.82, 1.34)	0.96 (0.74, 1.23)	0.99 (0.77, 1.28)	1.01 (0.93, 1.09)	0.856
Model 2	1 (ref)	1.11 (0.86, 1.43)	1.13 (0.87, 1.45)	1.02 (0.78, 1.32)	0.99 (0.76, 1.30)	0.99 (0.91, 1.08)	0.795
Fiber from vegetables							
Case number	142	129	143	118	103		
Model 1	1 (ref)	0.90 (0.71, 1.14)	0.99 (0.79, 1.26)	0.82 (0.65, 1.05)	0.72 (0.56, 0.93)	0.85 (0.76, 0.95)	0.004
Model 2	1 (ref)	0.92 (0.72, 1.17)	0.98 (0.77, 1.24)	0.75 (0.58, 0.98)	0.58 (0.43, 0.77)	0.76 (0.67, 0.86)	<0.001
Fiber from beans							
Case number	133	132	130	112	128		
Model 1	1 (ref)	0.98 (0.77, 1.25)	0.98 (0.77, 1.25)	0.84 (0.65, 1.08)	0.96 (0.75, 1.23)	0.95 (0.86, 1.05)	0.308
Model 2	1 (ref)	1.02 (0.80, 1.30)	1.01 (0.79, 1.30)	0.84 (0.65, 1.09)	0.87 (0.66, 1.13)	0.88 (0.79, 0.98)	0.021
Fiber from grains							
Case number	135	130	120	129	121		
Model 1	1 (ref)	0.94 (0.74, 1.19)	0.86 (0.67, 1.10)	0.91 (0.72, 1.16)	0.84 (0.66, 1.07)	0.94 (0.86, 1.03)	0.200
Model 2	1 (ref)	0.93 (0.73, 1.19)	0.84 (0.65, 1.09)	0.88 (0.68, 1.15)	0.77 (0.58, 1.02)	0.92 (0.83, 1.01)	0.089

The SDs of intake were 0.86 oz/day for whole grains, 10.9 g/day for total dietary fiber, 3.6 g/day for fiber from fruits, 4.8 g/day for fiber from vegetables, 2.9 g/day for fiber from beans, 4.0 g/day for fiber from grains, and 2.4 g/day for fiber from cereals.

Cox proportional hazard regression models were used. All P-values were two-sided. Model 1 were stratified by sex, adjusted for age at baseline (continuous). Model 2 further adjusted for level of education ('<=11 yrs', 'High school', 'Vocational technology school', 'Some College', 'College/Post Graduate'), race ('Non-Hispanic White', 'Non-Hispanic Black', 'Hispanic', 'Asian, Pacific Islander, or American Indian/Alaskan Native'), BMI ('<25', '25-<30', '30+', kg/m^2), alcohol use ('Non-drinker', '0.1-4.9', '5-9.9', '10+',gram/day), tobacco smoking ('Never smoked', 'Former smoker', 'Current smoker'), physical activity ('Never', 'Rarely', '1-3 time per month', '1-2 times per week', '3-4 times per week', '5+ times per week'), history of diabetes ('No', 'Yes') and total energy intake (continuous).

Supplementary Table 6 The association between whole grains and dietary fiber with risk of ICC among participants of NIH-AARP Diet and Health Study

	HR (95% CI)						*P trend*
	Quintile 1	Quintile 2	Quintile 3	Quintile 4	Quintile 5	Per SD increase	
Whole grains							
Case number	33	39	34	29	29		
Model 1	1 (ref)	1.16 (0.73, 1.84)	0.97 (0.60, 1.56)	0.84 (0.51, 1.38)	0.82 (0.50, 1.36)	0.91 (0.76, 1.08)	0.286
Model 2	1 (ref)	1.15 (0.72, 1.83)	0.98 (0.60, 1.59)	0.86 (0.51, 1.43)	0.86 (0.51, 1.46)	0.93 (0.77, 1.12)	0.427
Total dietary fiber							
Case number	30	38	33	29	34		
Model 1	1 (ref)	1.24 (0.77, 2.00)	1.06 (0.65, 1.74)	0.92 (0.55, 1.54)	1.08 (0.66, 1.76)	0.95 (0.78, 1.15)	0.590
Model 2	1 (ref)	1.27 (0.78, 2.08)	1.11 (0.65, 1.88)	0.98 (0.55, 1.74)	1.20 (0.64, 2.24)	0.97 (0.74, 1.26)	0.802
Fiber from fruits							
Case number	37	35	28	29	35		
Model 1	1 (ref)	0.90 (0.57, 1.43)	0.71 (0.43, 1.16)	0.72 (0.44, 1.17)	0.86 (0.54, 1.37)	1.02 (0.87, 1.20)	0.811
Model 2	1 (ref)	0.91 (0.57, 1.45)	0.72 (0.44, 1.20)	0.74 (0.45, 1.23)	0.91 (0.55, 1.51)	1.06 (0.89, 1.26)	0.520
Fiber from vegetables							
Case number	30	33	32	41	28		
Model 1	1 (ref)	1.09 (0.67, 1.79)	1.05 (0.64, 1.73)	1.35 (0.84, 2.16)	0.93 (0.55, 1.55)	0.94 (0.77, 1.15)	0.555
Model 2	1 (ref)	1.10 (0.67, 1.82)	1.07 (0.64, 1.79)	1.38 (0.83, 2.29)	0.96 (0.53, 1.71)	0.94 (0.74, 1.18)	0.570
Fiber from beans							
Case number	30	32	37	20	45		
Model 1	1 (ref)	1.05 (0.64, 1.73)	1.23 (0.76, 2.00)	0.66 (0.38, 1.17)	1.50 (0.94, 2.38)	1.06 (0.90, 1.25)	0.473
Model 2	1 (ref)	1.06 (0.64, 1.75)	1.26 (0.78, 2.06)	0.69 (0.39, 1.24)	1.64 (0.99, 2.72)	1.08 (0.91, 1.29)	0.371
Fiber from grains							
Case number	28	42	30	36	28		
Model 1	1 (ref)	1.47 (0.91, 2.38)	1.04 (0.62, 1.74)	1.24 (0.76, 2.03)	0.95 (0.56, 1.61)	0.89 (0.74, 1.07)	0.198
Model 2	1 (ref)	1.47 (0.91, 2.39)	1.05 (0.62, 1.80)	1.27 (0.75, 2.17)	1.01 (0.56, 1.82)	0.89 (0.71, 1.10)	0.273

The SDs of intake were 0.86 oz/day for whole grains, 10.9 g/day for total dietary fiber, 3.6 g/day for fiber from fruits, 4.8 g/day for fiber from vegetables, 2.9 g/day for fiber from beans, 4.0 g/day for fiber from grains, and 2.4 g/day for fiber from cereals.

Cox proportional hazard regression models were used. All P-values were two-sided. Model 1 were stratified by sex, adjusted for age at baseline (continuous). Model 2 further adjusted for level of education ('<=11 yrs', 'High school', 'Vocational technology school', 'Some College', 'College/Post Graduate'), race ('Non-Hispanic White', 'Non-Hispanic Black', 'Hispanic', 'Asian, Pacific Islander, or American Indian/Alaskan Native'), BMI ('<25', '25-<30', '30+', kg/m^2), alcohol use ('Non-drinker', '0.1-4.9', '5-9.9', '10+',gram/day), tobacco smoking ('Never smoked', 'Former smoker', 'Current smoker'), physical activity ('Never', 'Rarely', '1-3 time per month', '1-2 times per week', '3-4 times per week', '5+ times per week'), history of diabetes ('No', 'Yes') and total energy intake (continuous).

Supplementary Table 7 The association between whole grains and dietary fiber intake with CLD mortality among male participants of NIH-AARP Diet and Health Study

	HR (95% CI)						*P trend*
	Quintile 1	Quintile 2	Quintile 3	Quintile 4	Quintile 5	Per SD increase	
Whole grains							
Case number	213	163	129	104	83		
Model 1	1 (ref)	0.74 (0.61, 0.91)	0.56 (0.45, 0.70)	0.46 (0.37, 0.59)	0.36 (0.28, 0.47)	0.68 (0.62, 0.75)	<0.001
Model 2	1 (ref)	0.83 (0.67, 1.02)	0.66 (0.53, 0.82)	0.55 (0.43, 0.70)	0.42 (0.33, 0.55)	0.73 (0.66, 0.81)	<0.001
Total dietary fiber							
Case number	207	147	142	93	103		
Model 1	1 (ref)	0.69 (0.56, 0.86)	0.66 (0.53, 0.82)	0.43 (0.33, 0.55)	0.47 (0.37, 0.60)	0.68 (0.61, 0.76)	<0.001
Model 2	1 (ref)	0.67 (0.54, 0.84)	0.61 (0.49, 0.77)	0.38 (0.29, 0.49)	0.38 (0.28, 0.51)	0.61 (0.53, 0.70)	<0.001
Fiber from fruits							
Case number	175	136	132	122	127		
Model 1	1 (ref)	0.73 (0.59, 0.92)	0.70 (0.56, 0.87)	0.63 (0.50, 0.80)	0.65 (0.52, 0.82)	0.86 (0.78, 0.94)	0.001
Model 2	1 (ref)	0.89 (0.71, 1.11)	0.92 (0.73, 1.17)	0.90 (0.71, 1.14)	0.98 (0.77, 1.25)	0.99 (0.90, 1.08)	0.798
Fiber from vegetables							
Case number	170	152	141	125	104		
Model 1	1 (ref)	0.89 (0.71, 1.11)	0.82 (0.66, 1.03)	0.73 (0.58, 0.92)	0.61 (0.48, 0.78)	0.78 (0.70, 0.87)	<0.001
Model 2	1 (ref)	0.90 (0.72, 1.12)	0.81 (0.65, 1.03)	0.70 (0.54, 0.89)	0.55 (0.42, 0.73)	0.74 (0.65, 0.84)	<0.001
Fiber from beans							
Case number	142	157	156	118	119		
Model 1	1 (ref)	1.09 (0.87, 1.37)	1.10 (0.88, 1.38)	0.83 (0.65, 1.06)	0.84 (0.66, 1.07)	0.95 (0.87, 1.04)	0.264
Model 2	1 (ref)	1.10 (0.88, 1.38)	1.08 (0.86, 1.36)	0.80 (0.62, 1.03)	0.78 (0.60, 1.02)	0.94 (0.85, 1.04)	0.201
Fiber from grains							
Case number	229	157	133	87	86		
Model 1	1 (ref)	0.67 (0.55, 0.82)	0.56 (0.45, 0.70)	0.36 (0.28, 0.47)	0.35 (0.28, 0.45)	0.65 (0.59, 0.72)	<0.001
Model 2	1 (ref)	0.64 (0.52, 0.78)	0.51 (0.41, 0.64)	0.31 (0.24, 0.41)	0.29 (0.22, 0.38)	0.61 (0.54, 0.68)	<0.001

Cox proportional hazard regression models were used. All P-values were two-sided. Model 1 were adjusted for age at baseline (continuous). Model 2 further adjusted for level of education ('<=11 yrs', 'High school', 'Vocational technology school', 'Some College', 'College/Post Graduate'), race ('Non-Hispanic White', 'Non-Hispanic Black', 'Hispanic', 'Asian, Pacific Islander, or American Indian/Alaskan Native'), BMI ('<25', '25-<30', '30+', kg/m^2), alcohol use ('Non-drinker', '0.1-4.9', '5-9.9', '10+',gram/day), tobacco smoking ('Never smoked', 'Former smoker', 'Current smoker'), physical activity ('Never', 'Rarely', '1-3 time per month', '1-2 times per week', '3-4 times per week', '5+ times per week'), history of diabetes ('No', 'Yes') and total energy intake (continuous).

Supplementary Table 8 The association between whole grains and dietary fiber intake and CLD mortality among female participants of NIH-AARP Diet and Health Study

	HR (95% CI)						*P trend*
	Quintile 1	Quintile 2	Quintile 3	Quintile 4	Quintile 5	Per SD increase	
Whole grains							
Case number	97	57	67	37	43		
Model 1	1 (ref)	0.58 (0.42, 0.81)	0.66 (0.48, 0.90)	0.37 (0.25, 0.53)	0.42 (0.29, 0.60)	0.63 (0.52, 0.75)	<0.001
Model 2	1 (ref)	0.64 (0.46, 0.89)	0.74 (0.54, 1.02)	0.42 (0.28, 0.62)	0.47 (0.32, 0.69)	0.67 (0.55, 0.81)	<0.001
Total dietary fiber							
Case number	98	67	55	37	44		
Model 1	1 (ref)	0.67 (0.49, 0.91)	0.54 (0.39, 0.76)	0.36 (0.25, 0.53)	0.43 (0.30, 0.61)	0.63 (0.53, 0.76)	<0.001
Model 2	1 (ref)	0.67 (0.48, 0.92)	0.53 (0.37, 0.76)	0.34 (0.22, 0.52)	0.36 (0.22, 0.58)	0.60 (0.47, 0.76)	<0.001
Fiber from fruits							
Case number	71	67	59	57	47		
Model 1	1 (ref)	0.90 (0.64, 1.25)	0.78 (0.55, 1.10)	0.74 (0.52, 1.04)	0.60 (0.41, 0.87)	0.86 (0.74, 0.98)	0.029
Model 2	1 (ref)	1.12 (0.80, 1.57)	1.09 (0.77, 1.56)	1.14 (0.79, 1.64)	1.01 (0.67, 1.51)	1.04 (0.90, 1.19)	0.632
Fiber from vegetables							
Case number	80	66	66	44	45		
Model 1	1 (ref)	0.82 (0.59, 1.13)	0.81 (0.59, 1.13)	0.54 (0.38, 0.78)	0.56 (0.39, 0.81)	0.81 (0.69, 0.95)	0.011
Model 2	1 (ref)	0.84 (0.61, 1.18)	0.84 (0.60, 1.18)	0.55 (0.37, 0.81)	0.55 (0.36, 0.85)	0.83 (0.69, 1.00)	0.049
Fiber from beans							
Case number	81	73	53	57	37		
Model 1	1 (ref)	0.89 (0.65, 1.22)	0.65 (0.46, 0.92)	0.71 (0.50, 0.99)	0.46 (0.31, 0.68)	0.72 (0.57, 0.91)	0.006
Model 2	1 (ref)	0.90 (0.66, 1.24)	0.68 (0.48, 0.97)	0.71 (0.50, 1.02)	0.46 (0.30, 0.70)	0.74 (0.58, 0.95)	0.017
Fiber from grains							
Case number	99	73	53	40	36		
Model 1	1 (ref)	0.73 (0.54, 0.99)	0.52 (0.38, 0.73)	0.39 (0.27, 0.56)	0.35 (0.24, 0.51)	0.57 (0.47, 0.68)	<0.001
Model 2	1 (ref)	0.71 (0.52, 0.96)	0.49 (0.34, 0.69)	0.35 (0.24, 0.52)	0.29 (0.19, 0.45)	0.53 (0.42, 0.65)	<0.001

Cox proportional hazard regression models were used. All P-values were two-sided. Model 1 were adjusted for age at baseline (continuous). Model 2 further adjusted for level of education ('<=11 yrs', 'High school', 'Vocational technology school', 'Some College', 'College/Post Graduate'), race ('Non-Hispanic White', 'Non-Hispanic Black', 'Hispanic', 'Asian, Pacific Islander, or American Indian/Alaskan Native'), BMI ('<25', '25-<30', '30+', kg/m^2), alcohol use ('Non-drinker', '0.1-4.9', '5-9.9', '10+',gram/day), tobacco smoking ('Never smoked', 'Former smoker', 'Current smoker'), physical activity ('Never', 'Rarely', '1-3 time per month', '1-2 times per week', '3-4 times per week', '5+ times per week'), history of diabetes ('No', 'Yes') and total energy intake (continuous).

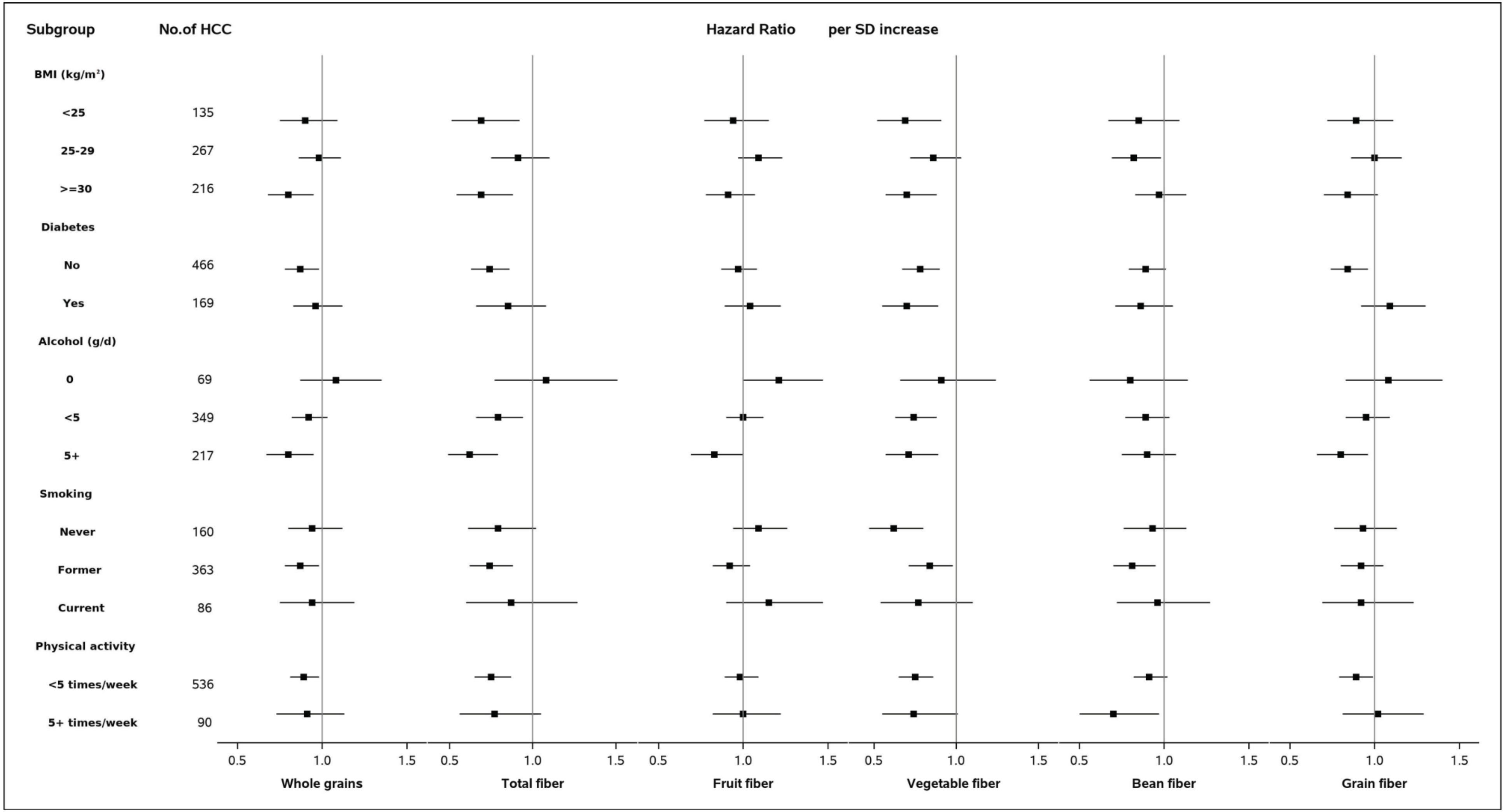

Supplementary Figure 1 Stratified analyses for association between whole grains and dietary fiber intake with risk of HCC among the participants of NIH-AARP Diet and Health Study.

Cox proportional hazard regression models were used. All P-values were two-sided. Models were stratified by sex and adjusted for age at baseline (continuous), level of education ('<=11 years', 'High school', 'Vocational technology school ', 'Some College', 'College/Post Graduate'), race ('Non-Hispanic White', 'Non-Hispanic Black', 'Hispanic', 'Asian, Pacific Islander, or American Indian/Alaskan Native'), BMI ('<25', '25-<30', '30+', kg/m^2), alcohol use ('Non-drinker', '0.1-4.9', '5-9.9', '10+', gram/day), tobacco smoking ('Never smoked', 'Former smoker', 'Current smoker'), physical activity ('Never', 'Rarely', '1-3 time per month', '1-2 times per week', '3-4 times per week', '5 + times per week'), history of diabetes ('No', 'Yes') and total energy intake (continuous), except when the variable is used for stratification.

Abbreviations: BMI: body mass index; SD: standard deviation; NIH-AARP: National Institutes of Health–American Association of Retired Persons.
