# Supplementary material for: Higher intake of whole grains and dietary fiber are associated with lower risk of liver cancer and chronic liver disease mortality
Source: Nat Commun. 2021 Nov 4;12:6388. doi: 10.1038/s41467-021-26448-9 (PMC8568891; doi:10.1038/s41467-021-26448-9)
Supplement: Supplementary file 2 — Reporting Summary [file 41467_2021_26448_MOESM2_ESM.pdf]

nature portfolio

Corresponding author(s): Xuehong Zhang

Last updated by author(s): Sep 8, 2021

Reporting Summary

Nature Portfolio wishes to improve the reproducibility of the work that we publish. This form provides structure for consistency and transparency in reporting. For further information on Nature Portfolio policies, see our Editorial Policies and the Editorial Policy Checklist.

Statistics

For all statistical analyses, confirm that the following items are present in the figure legend, table legend, main text, or Methods section.

n/a	Confirmed	
☐	☒	The exact sample size (*n*) for each experimental group/condition, given as a discrete number and unit of measurement
☐	☒	A statement on whether measurements were taken from distinct samples or whether the same sample was measured repeatedly
☐	☒	The statistical test(s) used AND whether they are one- or two-sided<br>*Only common tests should be described solely by name; describe more complex techniques in the Methods section.*
☐	☒	A description of all covariates tested
☐	☒	A description of any assumptions or corrections, such as tests of normality and adjustment for multiple comparisons
☐	☒	A full description of the statistical parameters including central tendency (e.g. means) or other basic estimates (e.g. regression coefficient) AND variation (e.g. standard deviation) or associated estimates of uncertainty (e.g. confidence intervals)
☐	☒	For null hypothesis testing, the test statistic (e.g. *F*, *t*, *r*) with confidence intervals, effect sizes, degrees of freedom and *P* value noted<br>*Give P values as exact values whenever suitable.*
☒	☐	For Bayesian analysis, information on the choice of priors and Markov chain Monte Carlo settings
☒	☐	For hierarchical and complex designs, identification of the appropriate level for tests and full reporting of outcomes
☐	☒	Estimates of effect sizes (e.g. Cohen's *d*, Pearson's *r*), indicating how they were calculated

Our web collection on statistics for biologists contains articles on many of the points above.

Software and code

Policy information about availability of computer code

Data collection	No software was used.
Data analysis	SAS 9.4 software was used for all analyses (SAS Institute Inc., Cary, NC, USA).

For manuscripts utilizing custom algorithms or software that are central to the research but not yet described in published literature, software must be made available to editors and reviewers. We strongly encourage code deposition in a community repository (e.g. GitHub). See the Nature Portfolio guidelines for submitting code & software for further information.

Data

Policy information about availability of data

All manuscripts must include a data availability statement. This statement should provide the following information, where applicable:

- Accession codes, unique identifiers, or web links for publicly available datasets
- A description of any restrictions on data availability
- For clinical datasets or third party data, please ensure that the statement adheres to our policy

Data and code are maintained by the National Cancer Institute, Division of Cancer Epidemiology and Genetics and are available upon submitting a proposal to be approved by the NIH-AARP Steering Committee. For more information visit https://www.nihaarpstars.com/

Field-specific reporting

Please select the one below that is the best fit for your research. If you are not sure, read the appropriate sections before making your selection.

☐ Life sciences ☒ Behavioural & social sciences ☐ Ecological, evolutionary & environmental sciences

For a reference copy of the document with all sections, see nature.com/documents/nr-reporting-summary-flat.pdf

Behavioural & social sciences study design

All studies must disclose on these points even when the disclosure is negative.

Study description	Quantitative cohort
Research sample	A total of 566,398 persons (339,666 men and 226,732 women) returned the baseline questionnaire and were included in the final cohort of the NIH–AARP Diet and Health Study recruited men and women aged 50-71 years old from six U.S. states (California, Florida, Louisiana, New Jersey, North Carolina and Pennsylvania) and two metropolitan areas (Atlanta, GA and Detroit, MI) in 1995-1996.
Sampling strategy	The NIH–AARP Diet and Health Study recruited men and women aged 50-71 years old by mailing questionnaires to its 3.5 million members from six U.S. states (California, Florida, Louisiana, New Jersey, North Carolina and Pennsylvania) and two metropolitan areas (Atlanta, GA and Detroit, MI) in 1995-1996. A total of 566,398 persons (339,666 men and 226,732 women) returned the baseline questionnaire and were included in the final cohort. For this analyses, we conducted a power analyses calculating minimum detectable HR under the current sample size. With a significance level of 0.05, a target power of 0.8, a population of 566,398 persons and the development of 940 liver cancer cases, the minimum detectable relative risk for a protective factor comparing top vs. bottom quintile groups would be 0.75.
Data collection	A questionnaire (pen and paper) was used to collect information on demographic characteristics, dietary intake and health-related behaviors through mail. Liver cancer cases were identified by linkage with state cancer registries and deaths due to chronic liver diseases were identified by the National Death Index Plus.
Timing	The NIH-AARP study recruited and started following-up participants in 1995-1996, and outcomes for this analysis were ascertained through December 31, 2011.
Data exclusions	In this analysis, we excluded participants with: 1) prevalent cancer (n=50,118) at baseline; 2) extreme caloric intakes (<500, or >3,500 for women; <800, or >4,000 for men; n=29,983); and 3) participants who were diagnosed with liver cancer or died from CLD before their baseline survey questionnaires were scanned (n=580).
Non-participation	Because the outcomes were ascertained through cancer registry or the National Death Index, the follow-up was complete for all participants.
Randomization	Not applicable.

Reporting for specific materials, systems and methods

We require information from authors about some types of materials, experimental systems and methods used in many studies. Here, indicate whether each material, system or method listed is relevant to your study. If you are not sure if a list item applies to your research, read the appropriate section before selecting a response.

Materials & experimental systems

n/a	Involved in the study	
☒	☐	Antibodies
☒	☐	Eukaryotic cell lines
☒	☐	Palaeontology and archaeology
☒	☐	Animals and other organisms
☐	☒	Human research participants
☒	☐	Clinical data
☒	☐	Dual use research of concern

Methods

n/a	Involved in the study	
☒	☐	ChIP-seq
☒	☐	Flow cytometry
☒	☐	MRI-based neuroimaging

Human research participants

Policy information about studies involving human research participants

Population characteristics	The NIH–AARP Diet and Health Study recruited men and women aged 50-71 years old from six U.S. states (California, Florida, Louisiana, New Jersey, North Carolina and Pennsylvania) and two metropolitan areas (Atlanta, GA and Detroit, MI) in 1995-1996. A total of 566,398 persons (339,666 men and 226,732 women) returned the baseline questionnaire and were included in the final cohort.
Recruitment	The NIH–AARP Diet and Health Study recruited men and women aged 50-71 years old by mailing questionnaires to its 3.5 million members, and a total of 566,398 persons (339,666 men and 226,732 women) returned the baseline questionnaire and were included in the final cohort. Since it is a cohort study, the follow-up rate is more important in reducing selection bias. The outcomes of this study was ascertained from cancer registries and the National Death Index Plus. Thus, the follow-up was complete for this analysis.
Ethics oversight	The study was approved by the Special Studies Institutional Review Board of the U.S. National Cancer Institute. Informed consent has been obtained from all participants.

Note that full information on the approval of the study protocol must also be provided in the manuscript.
